# Exploration of the Pathogenesis and Treatment of Heart Failure

**DOI:** 10.31083/RCM52463

**Published:** 2026-10-16

**Authors:** Bihai Wen, Shasha Yang, Huibo Wang

**Affiliations:** ^1^Department of Cardiology, Affiliated Central People's Hospital, China Three Gorges University, 443003 Yichang, Hubei, China; ^2^Department of Cardiology, Yichang Central People's Hospital, 443003 Yichang, Hubei, China; ^3^Hubei Key Laboratory of Ischemic Cardiovascular Disease, Yichang Central People's Hospital, 443003 Yichang, Hubei, China; ^4^Hubei Provincial Clinical Research Center for Ischemic Cardiovascular Disease, Yichang Central People's Hospital, 443003 Yichang, Hubei, China

**Keywords:** heart failure, cardiovascular disease, heart failure, diastolic, heart failure, systolic, stroke volume, ventricular remodeling

## Abstract

Heart failure represents the terminal stage of various cardiac diseases; the global prevalence of heart failure has been steadily increasing and poses a significant public health burden. With ongoing advances in molecular biology, clinical epidemiology, and translational medicine, research on heart failure has progressed significantly. Thus, understanding of the pathogenesis of heart failure has expanded beyond traditional hemodynamic abnormalities to encompass multidimensional, interrelated mechanisms, including neurohumoral imbalance, myocardial remodeling (hypertrophy and fibrosis), energy metabolism disorders, epigenetic regulation, and gut microbiota dysbiosis. This review systematically summarizes recent key advances in elucidating heart failure pathogenesis and iterative updates to treatment strategies, drawing on fundamental and translational research in this field.

## 1. Introduction

Heart failure refers to structural or functional abnormalities of the heart that impair ventricular filling or ejection capacity, leading to a characteristic set of symptoms (such as dyspnea, fatigue, and limitation of physical activity) and signs (such as pulmonary rales and peripheral edema) [1,2]. It affects approximately 64 million people worldwide, characterized by high incidence rates, numerous complications, and poor quality of life. Due to factors such as population aging, its prevalence may show an upward trend [3]. The overall incidence of heart failure has stabilized or declined. However, statistics indicate that the incidence among individuals <55 years old and those >85 years old has either remained stable or increased, showing significant population differentiation. Furthermore, differences exist among heart failure subtypes: the incidence of heart failure with reduced ejection fraction (HFrEF) has decreased, while that of heart failure with preserved ejection fraction (HFpEF) has increased [4]. Heart failure remains one of the leading causes of morbidity and mortality worldwide, continuing to impose a significant burden on healthcare systems due to its high mortality and hospitalization rates [5,6]. Therefore, gaining a deeper understanding of the pathophysiology and treatment of heart failure is crucial for its prevention, diagnosis, and management.

## 2. Pathophysiology of Heart Failure

Heart failure is a complex clinical syndrome with intricate pathophysiological mechanisms. It arises not from a single pathological abnormality but from the interplay of multiple systems, cells, and signaling pathways. A thorough understanding of these complex mechanisms is fundamental to developing effective therapeutic strategies.

### 2.1 Neurohumoral Mechanisms

Under physiological conditions, the renin-angiotensin-aldosterone system (RAAS) plays a role in regulating blood pressure and water-salt balance. In heart failure, the RAAS remains persistently overactivated, shifting from a compensatory mechanism to a pathological driving force, and serves as a central pathway promoting myocardial remodeling and worsening cardiac function [7].

In patients with heart failure, insufficient cardiac output leads to inadequate renal perfusion. Consequently, reduced perfusion pressure is transmitted to the afferent arteriolar baroreceptors, stimulating renin release. Renin then cleaves angiotensinogen produced by the liver into angiotensin I, which is converted into angiotensin II by angiotensin-converting enzyme (ACE). Angiotensin II exerts potent vasoconstrictive effects, increasing cardiac afterload, thereby promoting myocardial remodeling and stimulating aldosterone release [8]. This further leads to water and sodium retention, increasing cardiac volume load. Additionally, the RAAS system interacts with the sympathetic nervous system. Activation of the RAAS promotes norepinephrine release, while sympathetic activation further stimulates the RAAS, creating a vicious cycle that collectively exacerbates myocardial injury [7].

### 2.2 Cardiac Remodeling

Cardiac remodeling is the core pathological process in which the structure, function, metabolism, and electrophysiology of the myocardium undergo chronic, progressive changes in response to injury or abnormal loading, involving multifaceted regulatory networks such as neurohumoral, inflammatory, and fibrotic mechanisms [9,10].

#### 2.2.1 Myocardial Cell Remodeling

Myocardial remodeling is characterized by myocardial hypertrophy, cell death, calcium homeostasis imbalance, and energy metabolism disorders, ultimately leading to irreversible damage to systolic and diastolic function.

In the early stages, pathological myocardial hypertrophy represents a compensatory response of the heart to increased stress or excessive neurohumoral activation, maintaining cardiac output by enhancing myocardial contractility. However, when pathological stimuli persist, hypertrophy gradually shifts from a compensatory to a decompensatory state, and the synergistic action of multiple signaling pathways drives the onset and progression of heart failure [11]. Persistent hypertrophy can lead to disorganization of sarcomere arrangement and interstitial fibrosis, resulting in increased myocardial stiffness [12]. At the same time, neurohumoral networks, inflammatory networks, and epigenetic regulation continue to drive the expression of hypertrophy-related genes. Furthermore, abnormalities in calcium cycling and energy metabolism intertwine to impair myocardial contractile function [13]. Furthermore, m6A methylation (METTL3) [14] and activation of the cGAS-STING pathway [15] create a vicious cycle, ultimately causing the heart to transition completely from a compensated state to a decompensated state, leading to systolic and diastolic dysfunction and eventually progressing to heart failure.

In heart failure, myocardial cell death does not follow a single pattern but rather constitutes a complex network formed by the interplay of various programmed cell death mechanisms, including apoptosis, necroptosis, and pyroptosis, which collectively drive myocardial injury and worsening cardiac function [16]. Apoptosis, as a classic form of programmed cell death, is primarily initiated through the death receptor pathway (exogenous) and the mitochondrial pathway (endogenous). The death receptor pathway is triggered by ligands such as Fas/FasL, which activate caspase-8 through the formation of a death-inducing signal complex (DISC), thereby initiating a cascade of events; the mitochondrial pathway, on the other hand, is activated by endogenous stressors such as oxidative stress or mechanical stretching and is regulated by Bcl-2 family proteins. The pro-apoptotic proteins Bcl-2-associated X protein (BAX)/Bcl-2 antagonist/killer 1 (BAK) mediate an increase in mitochondrial outer membrane permeability (MOMP), leading to the release of cytochrome c. Cytochrome c then assembles with Apaf-1, dATP, and pro-caspase-9 to form an apoptosome, which activates the caspase cascade, ultimately resulting in the orderly disintegration of the cell [17]. Necrotic apoptosis is a regulated form of necrosis triggered by inflammatory factors that activate the RIPK1–RIPK3–MLKL pathway, leading to cell lysis, release of cellular contents, and amplification of the local inflammatory response [18]. Under physiological conditions, cardiomyocytes exhibit a baseline level of autophagy, which clears damaged organelles and misfolded proteins, maintains cellular structural and functional stability, and prevents cell death. However, under conditions of excessive or uncompensated stress, this process can lead to uncontrolled degradation of key proteins and organelles, shifting from an adaptive protective mechanism to a pro-apoptotic mechanism [19]. Pyroptosis in cardiomyocytes is a form of programmed inflammatory cell death mediated by the inflammasome. Under pathological stimuli such as oxidative stress and hypoxia, CaMKIIδ is activated, which in turn activates the NLR family pyrin domain containing 3 (NLRP3) inflammasome, leading to the activation of caspase-1 and the cleavage of gasdermin D (GSDMD). The N-terminal fragment of GSDMD forms pores in the cell membrane, resulting in pyroptosis and the release of pro-inflammatory factors such as interleukin-1β (IL-1β) and IL-18, thereby exacerbating myocardial inflammation and injury [20,21]. The aforementioned death mechanisms do not exist in isolation but rather overlap and interrelate, collectively leading to progressive loss of cardiomyocytes, myocardial fibrosis, and irreversible deterioration of cardiac function.

In addition, abnormal calcium handling in cardiomyocytes leads to impaired excitation-contraction coupling. Dysfunction of the sarcoplasmic reticulum calcium release channel (RyR2) is a central mechanism underlying calcium handling abnormalities in HF [22]. The ultrastructural decoupling of the T-tubules (TT) from the sarcoplasmic reticulum (SR), CaMKII hyperphosphorylation [23,24], oxidative modifications by reactive oxygen species (ROS) and reactive nitrogen species (RNS) [25], and functional abnormalities in regulatory proteins such as FK506-binding protein 12.6 (FKBP12.6) [26] collectively contribute to RyR2 calcium leakage [27]. Concurrently, reduced activity of the calcium pump SERCA2a and mitochondrial calcium transport imbalance lead to cytosolic calcium overload, which not only inhibits contractile function but also induces oxidative stress and cell death, thereby exacerbating myocardial remodeling [28].

#### 2.2.2 Extracellular Matrix (ECM) Remodeling

As a key non-cellular scaffold that maintains cardiac muscle structure and function, dysregulation of myocardial ECM remodeling leads to excessive deposition of ECM proteins, resulting in myocardial stiffness, scar formation, and ultimately heart failure [29].

Matrix metalloproteinases (MMPs), as a family of zinc-dependent endopeptidases, are key regulators of myocardial ECM remodeling. Dysregulation of their function is a central mechanism underlying abnormal ECM remodeling and directly contributes to the pathological progression of cardiovascular diseases. The MMP family comprises more than 20 members, among which MMP-2 plays the most prominent role in cardiovascular pathology. Within the endoplasmic reticulum of cardiomyocytes, MMP-2 degrades junctophilin-2 (JPH-2), disrupting the anchoring of T-tubules to the sarcoplasmic reticulum and leading to impaired excitation-contraction coupling [30].

It is worth noting that the balance between MMPs and their endogenous inhibitors (TIMPs) is key to regulating ECM metabolism. When MMP activity increases or TIMP expression decreases, ECM degradation becomes abnormal: excessive early degradation leads to ventricular dilation; excessive late-stage reparative collagen deposition exacerbates fibrosis [31]. Concurrently, studies have demonstrated that early inhibition of MMP-2 activity reduces JPH-2 degradation, preserves the structural integrity of the sarcolemma-sarcoplasmic reticulum, and improves myocardial contractile function. This imbalance further disrupts ECM homeostasis and promotes the onset and progression of myocardial remodeling [32].

#### 2.2.3 Energy Metabolism Reprogramming

In adult cardiomyocytes, fatty acid oxidation (FAO) serves as the primary energy source, accounting for approximately 60%–70% of total adenosine triphosphate (ATP) production in the myocardium, while glycolysis plays only a minor role. This highly efficient aerobic metabolic pathway depends on the structural integrity of mitochondria and the normal functioning of the oxidative phosphorylation (OxPhos) system, and it forms the foundation for maintaining the heart’s continuous pumping function [33].

However, under pathological conditions, the transcriptional repressor HEY2 suppresses the expression of key transcriptional co-activators (such as PGC-1α and ERRα), leading to impaired transcription of their downstream target genes (such as CPT1A and CPT2) and a sharp decline in fatty acid oxidation output. Concurrently, a reduction in mitochondrial number, impaired cristae structure, and decreased oxidative phosphorylation efficiency indicate that the myocardial energy production mode has shifted from a high-efficiency to a low-efficiency state [34].

To compensate for the deficiency in FAO, cardiomyocytes compensatorily increase anaerobic glycolysis. However, the energy yield of glycolysis is extremely low (producing only 2 ATP per glucose molecule, whereas FAO can generate over 100 ATP), and it produces large amounts of lactate, leading to intracellular acidosis. This not only directly inhibits myocardial contractility but also further exacerbates energy metabolism disorders by inhibiting fatty acid utilization [35]. Concurrently, abnormalities in branched-chain amino acid (BCAA) metabolism further exacerbate energy metabolism disorders. The RNA-binding protein G-rich sequence factor 1 (GRSF1) is a key regulator of BCAA metabolic homeostasis: under physiological conditions, GRSF1 stabilizes branched chain keto acid dehydrogenase E1 subunit beta (BCKDHB) mRNA, promotes BCAA catabolism, and prevents their abnormal accumulation. However, in patients with heart failure and in animal models, GRSF1 expression is significantly downregulated, leading to the accumulation of BCAAs and their metabolic intermediates (BCKAs) in the myocardium [36]. Furthermore, the accumulation of BCAAs not only promotes pathological myocardial hypertrophy and increases myocardial energy demand through leucine-activated mTOR signaling [37], but their metabolic intermediates, BCKAs, can also inhibit the activity of pyruvate dehydrogenase (PDH), preventing pyruvate produced by glycolysis from entering the mitochondria for complete oxidation and forcing the myocardium to rely more heavily on inefficient anaerobic glycolysis. Consequently, the BCAA metabolic disorder induced by GRSF1 downregulation intertwines with mitochondrial dysfunction, ultimately triggering myocardial remodeling and driving the progression of heart failure.

As the site where FAO, glucose oxidation, and OxPhos occur, mitochondrial dysfunction is both a direct consequence of the aforementioned metabolic disturbances and a key driver of heart failure progression [38]. Imbalances in mitochondrial Ca^2+^ homeostasis are a critical component of this process: in heart failure, abnormalities in mitochondrial Ca^2+^ transport channels and MAM-mediated calcium transport lead to mitochondrial Ca^2+^ overload. On the one hand, calcium overload causes energy metabolism disorders, massive production of ROS, and induces the opening of the mitochondrial permeability transition pore (mPTP), triggering myocardial cell apoptosis/necrosis [39]. On the other hand, calcium overload enhances the activity of mitochondrial fission proteins (such as Drp1) and reduces the expression of fusion proteins (such as Mfn1/2), leading to mitochondrial fragmentation. This hinders the formation of an efficient reticular energy supply network, and fragmented mitochondria are more susceptible to autophagy-mediated clearance, further impairing energy production [40]. Furthermore, calcium overload exacerbates myocardial inflammation and remodeling by increasing mtROS and mtDNA release, thereby activating inflammatory pathways such as the NLRP3 inflammasome and nuclear factor-kappa B (NF-κB) [41]. In summary, mitochondrial dysfunction and energy metabolism disorders are interrelated and mutually reinforcing, forming a vicious cycle that accelerates myocardial energy depletion and cardiac dysfunction. Targeting the regulation of mitochondrial calcium transport mechanisms represents a potential therapeutic strategy for heart failure.

### 2.3 Inflammation and Immune Mechanisms

In most cases, the activation of the inflammatory response serves as a reparative or protective reaction to tissue damage; however, excessive or dysregulated inflammation can exacerbate myocardial damage, thereby promoting the onset and progression of heart failure [42]. Systemic inflammation is widely recognized as a common pathological feature of both chronic and acute heart failure [43]. Furthermore, studies have shown that inflammation is closely associated with the onset, progression, and complications of heart failure [44].

During heart failure, levels of pro-inflammatory cytokines such as tumor necrosis factor-α (TNF-α) and interleukin-1 (IL-1) are significantly elevated both locally in the myocardium and systemically in the circulation [45,46]. In particular, TNF-α expression is increased in cardiomyocytes, macrophages, vascular endothelial cells, and mast cells of the failing heart [47]. TNF-α exerts a negative inotropic effect by inhibiting sarcoplasmic reticulum calcium ATPase activity and promoting excessive phosphorylation of the ryanodine receptor (RyR2), thereby directly impairing contractile function [47,48]. Concurrently, it accelerates cardiomyocyte apoptosis by activating the Bcl-2-related X protein (Bax)/Bcl-2 antagonist/killer 1 (Bak)-mediated intrinsic apoptotic pathway, thereby exacerbating cardiomyocyte apoptosis and reducing the number of surviving cardiomyocytes [49,50]. Furthermore, TNF-α expressed by macrophages stimulates the synthesis of other pro-inflammatory cytokines, which possess pro-apoptotic, negative inotropic, and matrix-degrading properties [51]. In addition to TNF-α, members of the interleukin family also play a key role in the onset and progression of heart failure. Studies have shown that IL-1 expression levels are elevated in various heart failure models and are often accompanied by inflammasome activation [52,53]. The inflammasome promotes the processing of IL-1β into its active form, which subsequently exerts pathogenic effects on myocardial tissue [54]. Similar to TNF-α, IL-1 exerts a negative inotropic effect and induces cardiomyocyte apoptosis [55,56].

In addition to inflammatory mediators, immune cells also play a key regulatory role in heart failure. Among CD4+ T cells, the Th1 subset secretes interferon-γ (IFN-γ), which promotes myocardial fibrosis [57]; the Th2 subset secretes interleukin-4 (IL-4), interleukin-13 (IL-13), and other substances that, while capable of suppressing inflammation, may also exacerbate fibrosis [58]. Furthermore, regulatory T cells (Tregs) secrete interleukin-10 (IL-10), which can alleviate inflammatory responses [59]. However, studies have shown that patients with heart failure have reduced numbers of Tregs and impaired Treg function, leading to immune dysregulation [60] (Fig. 1).

**Fig. 1.**
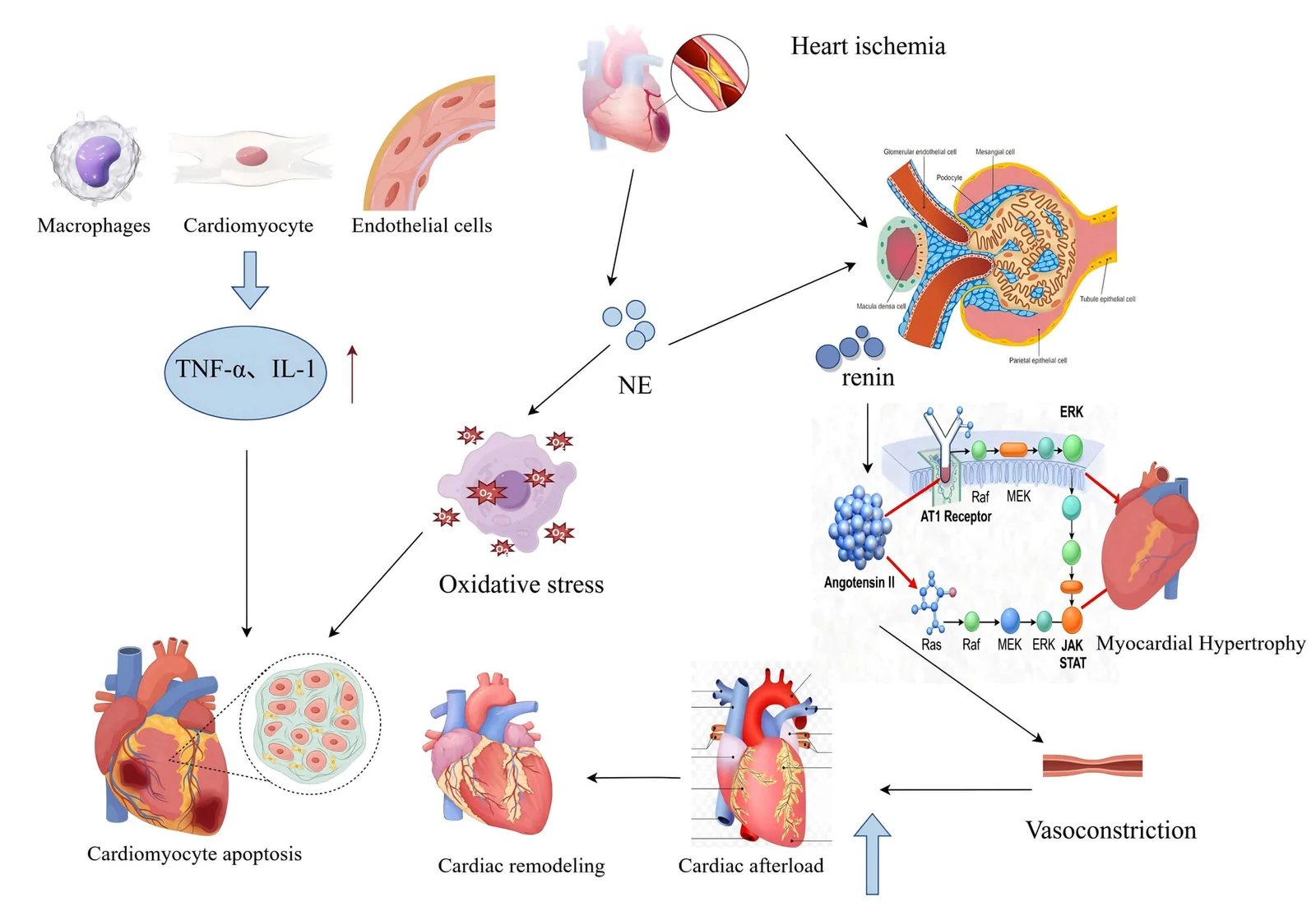
**Some pathological mechanisms of heart failure**. TNF-α, tumor necrosis factor-α; IL-1, interleukin-1; NE, norepinephrine; ERK, extracellular signal-regulated kinase.

### 2.4 Other Mechanisms

Recent studies have revealed that AngII can also mediate the recruitment of bone marrow-derived CXCR2^+^ monocytes to the heart via the C-X-C chemokine ligand 1 (CXCL1)/C-X-C chemokine receptor 2 (CXCR2) axis by upregulating CXCL1 and CXCR2. This process activates inflammation-related signaling pathways, leading to myocardial hypertrophy and fibrosis, which in turn impair cardiac contractile function; the CXCL1-CXCR2 axis plays a central mediating role throughout this process [61,62].

Gut microbiota dysbiosis can influence cardiac function through metabolic byproducts [63]. Trimethylamine N-oxide (TMAO) promotes macrophage infiltration and induces oxidative stress [64], whereas short-chain fatty acids (SCFAs) exert anti-inflammatory effects by inhibiting histone deacetylases (HDACs) [65]. Furthermore, epigenetic modifications (e.g., DNA methylation, RNA methylation) play crucial roles in metabolic memory and immune regulation [66]. For instance, METTL4-mediated N6-methyladenine modification of mitochondrial DNA contributes to the pathogenesis of heart failure [67]. Methyltransferase-like protein 14 (METTL14) promotes vascular endothelial cell senescence by modifying Toll-like receptor 4 (TLR4) mRNA, whereas inhibition of METTL14 restores vascular elasticity in aged mice [68].

## 3. Classification of Heart Failure

Heart failure represents the end stage of various cardiovascular diseases. The classification system based on left ventricular ejection fraction (LVEF) is currently recognized by international guidelines and is the most commonly used clinical classification standard; it effectively distinguishes subtypes of heart failure with different pathophysiological characteristics, prognoses, and treatment responses. A precise understanding of LVEF reference standards, measurement errors, and the clinical significance of each subtype is a prerequisite for the accurate diagnosis and individualized management of heart failure.

### 3.1 Normal Range of LVEF

LVEF is the percentage ratio of left ventricular stroke volume to end-diastolic volume; it is a key quantitative indicator for assessing overall left ventricular systolic function and classifying types of heart failure. According to authoritative domestic and international guidelines for the diagnosis and treatment of heart failure, as well as data from large-scale population studies, the normal reference value for LVEF measured by echocardiography in healthy adults at rest is no less than 50%, with the commonly accepted clinical normal reference range being 55%–70% [69,70].

### 3.2 Intra-Observer and Inter-Observer Variability in Echocardiographic LVEF Measurements

Echocardiography is the preferred noninvasive imaging modality for measuring LVEF, assessing cardiac systolic function, and determining the subtype of heart failure in patients with heart failure. It offers real-time imaging, ease of use, the absence of ionizing radiation, and the ability to be performed at the bedside, making it a standard core criterion for clinical assessment of cardiac function. However, LVEF measurements are susceptible to interference from various factors, including instrument settings, imaging planes, the patient’s condition, and the operator’s clinical experience. Significant intra- and inter-observer measurement variability exists, which directly affects the accuracy of heart failure subtype classification [71,72].

Intra-observer variability refers to the differences in LVEF measurements obtained by the same physician for the same patient at different times or under different conditions. It primarily stems from subtle deviations in manual tracing of ventricular borders, differences in image frame selection, and fluctuations in patient position or heart rate. In routine clinical measurements, the fluctuation range is approximately 3%–5% [71]. Inter-observer variability refers to the numerical deviation in LVEF measurements of the same patient by physicians with different levels of seniority and experience; this variation is more pronounced, with fluctuations ranging from 5% to 8% [72]. To reduce variability and improve the reliability of classification, clinical guidelines recommend calculating LVEF using the standardized cut and two-plane Simpson method, with independent review by two or more physicians and the use of the average of multiple measurements, which can minimize measurement error.

### 3.3 Mainstream Classification of Heart Failure Based on LVEF

Based on LVEF classification criteria, heart failure can be divided into three categories: HFrEF (LVEF <40%) involves severe impairment of systolic function and is commonly associated with organic heart disease; heart failure with mildly reduced ejection fraction (HFmrEF) (41% ≤ LVEF ≤ 49%) represents an intermediate phenotype characterized by both systolic and diastolic abnormalities; HFpEF (LVEF ≥50%) involves essentially normal systolic function, with diastolic dysfunction being the primary feature [73].

#### 3.3.1 Pathogenesis of HFrEF

The core pathophysiological feature of HFrEF is a significant decline in myocardial contractile function, leading to a reduced left ventricular ejection fraction and inadequate cardiac output. This pathophysiological process is primarily driven by the interplay of myocardial cell damage, contractile dysfunction, disturbances in calcium homeostasis, excessive neuroendocrine activation, and energy metabolism disorders [74].

Conditions such as myocardial infarction, dilated cardiomyopathy, and viral myocarditis can lead to myocardial necrosis, apoptosis, and pyroptosis. Since cardiomyocytes are terminally differentiated cells, they cannot regenerate following extensive damage, which directly results in an irreversible reduction in contractile units and a significant decline in contractile force [74,75]. In surviving myocardial tissue, disorganized myofilament alignment and abnormal myosin expression further impair contractile capacity [76,77]; simultaneously, reduced SERCA2a activity and increased RyR2 calcium leakage lead to calcium homeostasis imbalance and excitation-contraction decoupling, resulting in a sustained decline in contractile efficiency [78,79].

In addition, persistent pathological activation of the sympathetic nervous system and the RAAS leads to the release of large amounts of catecholamines and angiotensin II, inducing myocardial hypertrophy, fibrosis, and increased preload and afterload, thereby creating a vicious cycle that accelerates the deterioration of cardiac function [80].

In terms of energy metabolism, the myocardium shifts from efficient fatty acid oxidation to inefficient glycolysis; simultaneously, mitochondrial structural damage and dysfunction lead to insufficient ATP production, further impairing contractile function [81].

Although a wealth of experimental data has been accumulated in this field, current research on energy metabolism in HFrEF remains primarily based on animal models and small-sample clinical observations. Due to significant variations in experimental conditions and heterogeneity in results, high-quality prospective clinical evidence remains insufficient, and the clinical translational value requires further validation.

#### 3.3.2 Pathogenesis of HFpEF

HFpEF is characterized primarily by impaired ventricular diastolic function, increased myocardial stiffness, and pulmonary congestion, with largely normal systolic function; it is commonly seen in older adults, obese individuals, and those with hypertension or metabolic disorders [82,83]. HFpEF is not merely a simple abnormality of left ventricular diastolic function, but rather a multifactorial, systemic syndrome with a core mechanism related to inflammation, involving the interaction of multiple factors [84,85].

The core pathological change in HFpEF is inflammation-mediated increased myocardial stiffness. Comorbidities such as obesity, diabetes, and hypertension induce a systemic pro-inflammatory state, reducing nitric oxide bioavailability and inhibiting the cyclic guanosine monophosphate (cGMP) pathway, which leads to increased stiffness in the myocardium and blood vessels. Furthermore, epicardial adipose tissue secretes inflammatory factors, which further exacerbate myocardial inflammation and interstitial fibrosis, reduce ventricular compliance, and cause abnormalities in diastolic relaxation and filling, ultimately leading to left atrial remodeling, pulmonary arterial hypertension, and even progression to right heart failure [86].

Abnormal calcium homeostasis is a key pathophysiological mechanism specific to HFpEF, primarily manifested as delayed diastolic calcium reuptake. Reduced activity of the sarcoplasmic reticulum calcium pump (SERCA2a) in cardiac myocytes leads to slowed calcium reuptake and delayed cytoplasmic calcium clearance, directly impairing myocardial relaxation; simultaneously, abnormal myosin phosphorylation further affects sarcomere relaxation, collectively resulting in impaired ventricular diastolic function and elevated end-diastolic pressure [79].

In summary, HFpEF is characterized by a core pathological cascade driven by inflammation and fibrosis, which leads to increased stiffness and impaired diastolic calcium clearance, forming a distinct phenotypic profile that differentiates it from HFrEF.

#### 3.3.3 Pathophysiology of HFmrEF

HFmrEF, as an independent intermediate phenotype between HFrEF and HFpEF, exhibits pathophysiological mechanisms characteristic of both types of heart failure: it features the mild myocardial damage and neuroendocrine activation seen in HFrEF, as well as the diastolic abnormalities and myocardial fibrosis observed in HFpEF. Overall, it represents a transitional, reversible state that is significantly influenced by comorbidities and treatment [87].

Mild impairment of systolic function is the core characteristic of HFmrEF. Compared with HFrEF, myocardial cell loss is less severe, primarily manifesting as myocardial hypertrophy, abnormal expression of contractile proteins, and mild disturbances in calcium homeostasis, leading to a mild decrease in contractility and an LVEF maintained within the 40%–50% range [88,89]. This damage is often caused by the long-term accumulation of chronic stress, ischemia, and low-grade inflammation. Although it does not reach the severity of HFrEF, it results in a marked reduction in systolic reserve and serves as a potential trigger for worsening cardiac function.

Diastolic dysfunction is also a key pathological feature of HFmrEF. Similar to HFpEF, patients exhibit myocardial fibrosis, increased ventricular stiffness, delayed diastole, and impaired filling; however, CMR tissue characterization indicates that the degree of fibrosis in HFmrEF is closer to that of HFrEF than to HFpEF, as reflected by native T1 relaxation times [89]. Concurrently, endothelial dysfunction and coronary microcirculatory disturbances further exacerbate diastolic abnormalities, leading to elevated end-diastolic pressure and symptoms associated with pulmonary congestion [90].

In addition, excessive neuroendocrine activation and comorbidities are key drivers of the progression of HFmrEF. The neuroendocrine system in this condition is moderately activated, falling between the levels observed in HFrEF and normal levels. Moderate activation of the sympathetic and RAAS systems is sufficient to promote myocardial fibrosis and apoptosis, driving the continued deterioration of systolic and diastolic function; comorbidities such as hypertension, diabetes, and atrial fibrillation accelerate disease progression and contribute to the transition of some patients to HFrEF [69].

Although current guidelines generally recognize HFmrEF as a distinct intermediate phenotype, the clinical validity of this classification remains controversial. Some researchers advocate for the elimination of HFmrEF as an official phenotypic classification, arguing that this classification lacks practical clinical value, is prone to misclassification due to measurement variability, and may lead to unnecessary medical interventions and potential iatrogenic risks [91]. Conversely, a growing body of evidence supports the independent existence of HFmrEF as a transitional state, characterized by unique pathophysiological features, prognostic significance, and treatment responses distinct from those of HFrEF and HFpEF [92,93] (Table 1).

**Table 1.** **Mechanism Comparison of HFrEF / HFpEF / HFmrEF**.

Type	HFrEF	HFpEF	HFmrEF
Underlying Pathology	Significant reduction in contractile function and decreased ejection capacity	Diastolic dysfunction, impaired ventricular filling	Mild systolic dysfunction combined with diastolic dysfunction
LVEF	Significantly reduced	normal/near normal	40% ≤ LVEF < 50%
Key pathophysiological mechanisms	Massive loss of cardiomyocytes (necrosis, apoptosis, pyroptosis)	Systemic inflammation → Increased stiffness of the myocardium and blood vessels	Mild cardiomyocyte damage + mild to moderate myocardial fibrosis

HFrEF, heart failure with reduced ejection fraction; HFpEF, heart failure with preserved ejection fraction; HFmrEF, heart failure with mildly reduced ejection fraction; LVEF, left ventricular ejection fraction.

## 4. Treatment of Chronic Heart Failure

Treatment strategies for chronic heart failure have evolved from simply addressing hemodynamic disturbances to a comprehensive management model centered on neuroendocrine antagonism, reversal of myocardial remodeling, and regulation of metabolism and inflammation. Classification based on LVEF remains a key basis for clinical decision-making [94]. However, existing evidence is primarily focused on HFrEF, and treatment for HFmrEF and HFpEF is often extrapolated from HFrEF, lacking specific evidence-based support, resulting in a significant gap in clinical evidence.

### 4.1 HFrEF: Centered on the Four-Pronged Therapy

The treatment of HFrEF focuses on improving prognosis, alleviating symptoms, and reducing mortality and the risk of hospitalization, encompassing both pharmacological and non-pharmacological interventions [73,88,95]. Currently, a four-drug regimen centered on angiotensin receptor-neprilysin inhibitor (ARNI), beta-blockers, mineralocorticoid receptor antagonists (MRA), and sodium-glucose cotransporter 2 inhibitors (SGLT2i) has become the cornerstone of pharmacological treatment for HFrEF [96,97]. Multiple large randomized controlled trials (RCTs) have demonstrated that this regimen significantly reduces all-cause mortality, cardiovascular mortality, and readmission rates for heart failure [98,99]. However, given the stringent inclusion criteria of these RCTs, the efficacy and safety of this regimen in real-world settings—particularly among patients with New York Heart Association (NYHA) Class IV heart failure, the elderly, or those with multiple organ dysfunction—remain to be further validated [100].

ARNI (such as sacubitril/valsartan) exerts vasodilatory effects and improves cardiac remodeling by inhibiting the excessive activation of the renin-angiotensin system and neuroendocrine pathways. Compared with traditional angiotensin-converting enzyme inhibitors (ACEIs) or angiotensin receptor blockers (ARBs), ARNI further reduces the risk of cardiovascular death and hospitalization for heart failure [101,102]. β-blockers slow the heart rate and reduce myocardial oxygen consumption by inhibiting excessive sympathetic nervous system activation; they are indicated for all patients with stable HFrEF. Multiple RCTs have demonstrated that they significantly improve clinical outcomes and reduce the risk of death [103,104]. MRA blocks the harmful effects of aldosterone on the heart, inhibits myocardial fibrosis and adverse remodeling, and thereby improves cardiac function [105,106]. SGLT2i promote the excretion of glucose and sodium in the urine, thereby exerting diuretic and antihypertensive effects and reducing both preload and afterload on the heart; they also improve myocardial energy metabolism and inhibit fibrosis and remodeling. In addition to reducing the risk of cardiovascular death and hospitalization for heart failure, SGLT2i can also slow the progression of kidney disease and improve quality of life [107,108].

For patients who do not respond well to medical therapy or are at high risk, device-based therapies may be considered: Cardiac resynchronization therapy (CRT) is indicated for patients with LVEF ≤35%, NYHA class II–IV heart failure, and a QRS width ≥130 ms. It corrects electromechanical dyssynchrony through biventricular pacing, improves systolic function, and reduces the risk of hospitalization for heart failure; Implantable cardioverter-defibrillators (ICDs) are indicated for patients with LVEF ≤35%, NYHA class II–III heart failure, and ischemic cardiomyopathy (≥40 days post-myocardial infarction) or non-ischemic cardiomyopathy (disease duration ≥3 months). ICDs detect and terminate malignant ventricular arrhythmias, thereby preventing sudden cardiac death [73]. It is worth noting that the clinical evidence for device therapy largely stems from RCTs conducted a decade ago; with advances in modern optimized pharmacological therapy, the additional benefits of such devices require reevaluation.

### 4.2 Treatment of HFmrEF

The clinical characteristics of patients with HFmrEF fall between those of HFrEF and HFpEF; treatment primarily follows the principles for HFrEF, but requires an individualized approach [73]. Currently, SGLT2i-based therapy combined with an MRA (such as finerenone) has become the core pharmacological strategy for HFmrEF, with finerenone shown to reduce the risk of heart failure hospitalization in this population [109]. Depending on comorbidities (such as obesity, diabetes, and hypertension), glucagon-like peptide-1 receptor agonists (GLP-1RAs) or ARNIs may be added; ARNI has been associated with improved outcomes in patients with HFmrEF and worsening heart failure [110], and GLP-1RAs have shown potential benefits in a recent meta-analysis [111]. Substantial evidence indicates that this combination regimen significantly reduces the risk of cardiovascular death and hospitalization for heart failure, while improving quality of life and prognosis [112]. However, much of this evidence stems from pre-specified subgroup analyses or post-hoc analyses; prospective RCTs specifically designed for the HFmrEF population remain scarce. Independent clinical trials are needed in the future to determine the optimal treatment strategy [113].

For patients with HFmrEF and concomitant coronary artery disease, the use of beta-blockers may be considered. In patients with acute myocardial infarction and mildly reduced ejection fraction, vasodilating beta-blockers were associated with a lower incidence of the composite endpoint of cardiac death, recurrent myocardial infarction, or heart failure hospitalization at 3 years [114]. Furthermore, studies have found that beta-blockers were independently associated with a lower risk of long-term all-cause mortality in patients with HFmrEF [115]. However, it should be noted that the 2021 European Society of Cardiology (ESC) guidelines do not explicitly recommend beta-blockers for patients with HFmrEF (then classified as HFpEF), partly due to a lack of RCT evidence specifically targeting this population. Furthermore, the guidelines do not confirm any clear benefit for patients with atrial fibrillation (AF). Given that the prevalence of AF among patients with HFmrEF is as high as 49.7% and is comparable to that in other HF subtypes, heart rate control is of critical importance for patients with concomitant AF [116]. Ivabradine reduces heart rate by inhibiting sinoatrial node conduction. Retrospective studies and systematic reviews suggest that ivabradine can reduce readmission rates and mortality in patients with heart failure, with more pronounced efficacy when used in combination with beta-blockers [117]. Verapamil can alleviate myocardial calcium overload primarily by blocking L-type calcium channels, and studies have demonstrated that it improves LVEF [118]. It should be noted that the evidence for the aforementioned heart rate-modulating drugs largely stems from retrospective or open-label studies, and their clinical value still requires validation through RCTs.

### 4.3 Treatment of HFpEF

The primary treatment goals for HFpEF are to alleviate symptoms, improve quality of life, and reduce the risk of hospitalization for heart failure. Currently, there is no standardized drug regimen, and treatment must be tailored to each patient’s phenotype and comorbidities. In recent years, SGLT2i have brought about breakthrough advancements in this field.

SGLT2i reduce cardiac workload by promoting the excretion of glucose and sodium in urine, inhibit myocardial fibrosis and remodeling, and improve myocardial energy metabolism. The EMPEROR-Preserved trial demonstrated that empagliflozin reduced the composite risk of cardiovascular death and hospitalization for heart failure by 21% in patients with HFpEF (regardless of diabetes status) and led to an early and sustained reduction in heart failure worsening events [119]. The DELIVER trial confirmed that dapagliflozin reduces the composite risk of heart failure worsening or cardiovascular death in patients with HFmrEF/HFpEF (LVEF >40%) [120].

Beyond these agents, medications such as mineralocorticoid receptor antagonists (MRAs), ARNIs, and GLP-1 receptor agonists (GLP-1RAs) may be selected based on patient phenotypes. MRAs can suppress aldosterone-mediated myocardial fibrosis and stiffness and improve diastolic function. Studies have shown that spironolactone can reduce the risk of cardiovascular death and hospitalization for heart failure in patients with LVEF levels of 45%–50% (near the lower limit for HFpEF) [121].

ARNI is indicated for patients with HFpEF who have an LVEF <57% or concomitant hypertension [122]. This drug simultaneously inhibits angiotensin receptors and kininase, thereby dilating blood vessels, reducing diastolic load on the heart, and inhibiting myocardial remodeling. Subgroup analyses from the Prospective Comparison of ARNI with ARB Global Outcomes in Heart Failure with Preserved Ejection Fraction (PARAGON-HF) trial demonstrated that sacubitril/valsartan reduced the risk of the composite endpoint by 22% in patients with HFpEF and LVEF <57%, while also improving renal function and NYHA class [123]. However, this drug did not show significant benefits in the overall HFpEF population, suggesting that its use should be based on LVEF-based selection.

As novel metabolic modulators, GLP-1 receptor agonists (GLP-1RAs) improve cardiac function and enhance clinical efficacy through multiple mechanisms, and have become a focal point of research in this field. In obese patients with HFpEF, semaglutide reduces the risk of cardiovascular death or heart failure worsening by 31% (HR = 0.69, 95% CI 0.53–0.89); tirzepatide reduces the risk of the composite endpoint by 38% (HR = 0.62) and significantly improves Kansas City cardiomyopathy questionnaire (KCCQ) scores and NT-proBNP levels [124,125,126]. These drugs are particularly suitable for obese patients with a body mass index (BMI) ≥30 kg/m^2^ or HFpEF patients with concomitant type 2 diabetes and have been classified as Class IIb recommendations by the European Society of Cardiology. However, their long-term cardiovascular outcomes and differences in efficacy across different HFpEF subtypes still require clarification through larger-scale studies.

## 5. Treatment of Acute Heart Failure

Acute heart failure (AHF) refers to a clinical syndrome caused by an acute episode of heart failure or a sudden exacerbation of pre-existing chronic heart failure, characterized by a sudden decrease in cardiac output and circulatory congestion. When complicated by cardiogenic shock, it is a critical condition with a short-term mortality rate as high as 40%–50% [127]. Treatment follows the principles of “stabilizing hemodynamics, correcting hypoxia, reducing congestion, maintaining organ perfusion, preventing complications, and addressing the underlying cause”, requiring an individualized and precise approach [73,128,129].

During the emergency management phase, immediate interventions include placing the patient in a semi-sitting position, administering oxygen therapy (maintaining SpO_2_ ≥95%), and establishing intravenous access. First-line pharmacotherapy involves diuretics [129]. When blood pressure exceeds 90 mmHg, vasodilators (nitrates or sodium nitroprusside) should be added. Morphine may be administered for severe dyspnea. If diuretics prove ineffective, doses may be increased or combination therapy initiated; ultrafiltration should be considered when necessary. Patients with hemodynamic instability (systolic blood pressure <90 mmHg with tissue hypoperfusion) require inotropic agents such as dobutamine, milrinone, or levosimendan. Persistent hypotension (<70 mmHg) may warrant vasopressors like norepinephrine.

New treatment approaches emphasize early initiation of SGLT2i (e.g., dapagliflozin) within 24–48 hours after stabilization [130,131]. For HFrEF patients, ARNI/ACEI/ARB and beta-blockers should be initiated upon stabilization. Additionally, non-pharmacological interventions include non-invasive ventilation (BiPAP), mechanical circulatory support (ECMO/Impella), and renal replacement therapy (when diuretic-resistant). Treatment strategies must be tailored to specific patient populations (e.g., elderly patients, patients with impaired renal function, and patients with acute coronary syndrome complicated by heart failure). Current guidelines are largely based on short-term clinical outcomes; future research should include more prospective RCTs targeting different phenotypes of AHF to optimize individualized treatment pathways (Fig. 2).

**Fig. 2.**
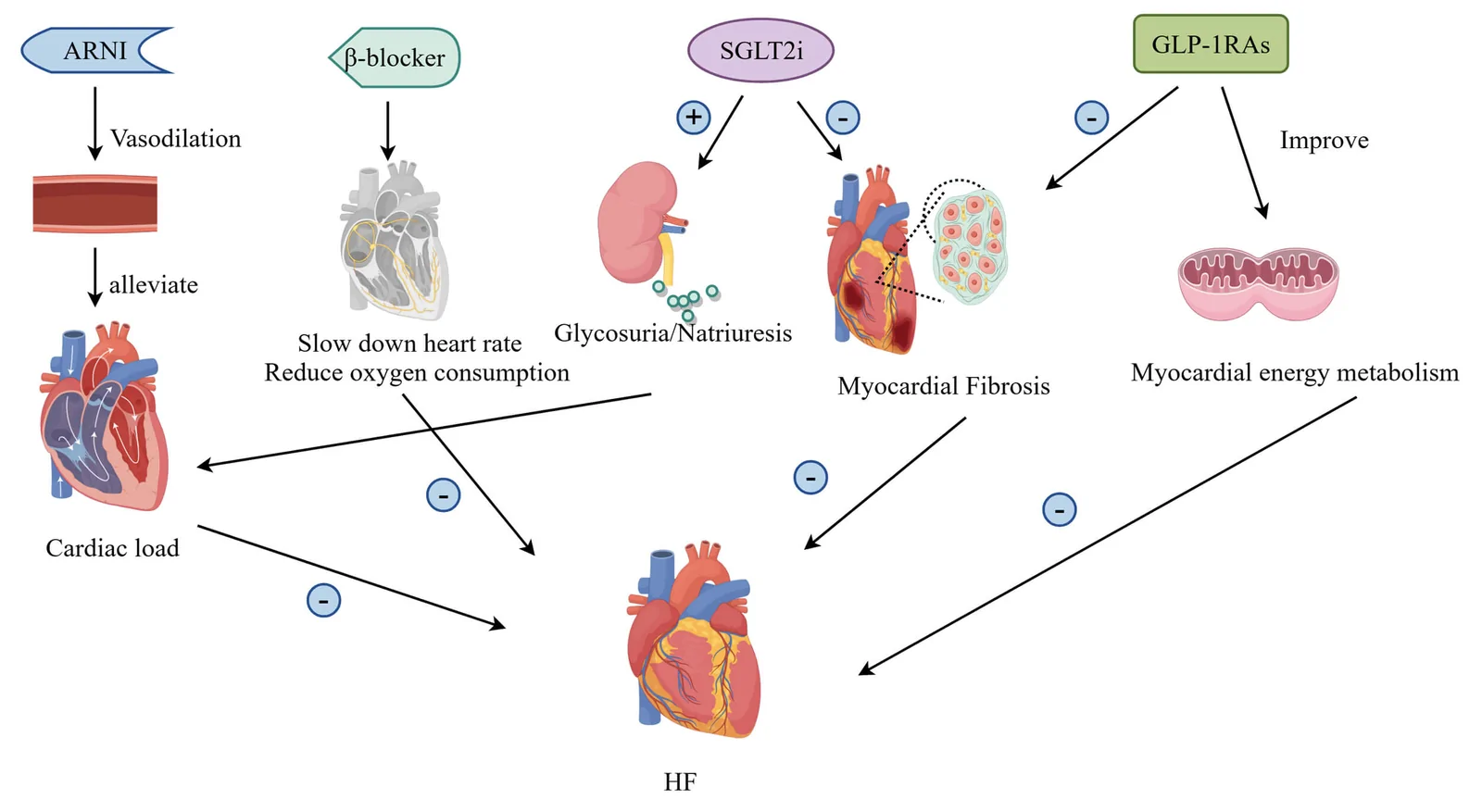
**Treatment of heart failure (HF)**. ARNI, angiotensin receptor–neprilysin inhibitors; SGLT2i, sodium-glucose cotransporter 2 inhibitors; GLP-1RAs, glucagon-like peptide-1 receptor agonists.

## 6. Gene Therapy for Heart Failure

While traditional medications (such as ACEIs and beta-blockers) can alleviate symptoms, they struggle to reverse molecular and structural damage to cardiomyocytes. Gene therapy directly targets core molecular pathological targets of heart failure. By precisely delivering therapeutic genes to myocardial tissue via vectors, it specifically repairs key pathological processes such as calcium homeostasis imbalance, myocardial remodeling, and fibrosis. Moreover, it avoids the potential systemic side effects of traditional drugs on non-target organs, achieving “precision-guided” treatment. Gene therapy for heart failure has progressed from concept to clinical reality, emerging as a novel strategy to expand current therapeutic approaches and intervene in previously inaccessible disease causative mechanisms. Currently, adeno-associated virus-based gene delivery is the mainstream technology [132], with targeted therapies focusing on key proteins such as sarcoplasmic reticulum calcium ATPase 2a (SERCA2a), phospholamban(PLN), and glycosylphosphatidylinositol-specific phospholipase D1.

As the most critical ion in the excitation-contraction coupling process of cardiomyocytes, disrupted calcium cycling represents one of the core pathological mechanisms of chronic heart failure (CHF), particularly the reduced activity of sarcoplasmic reticulum calcium ATPase 2a (SERCA2a) [133,134]. SERCA2a influences contraction and relaxation by regulating myocardial calcium homeostasis. Early animal studies demonstrated that SERCA2a gene therapy—achieved via direct plasmid injection, adenovirus vectors, adeno-associated virus (AAV) vectors (e.g., AAV1) demonstrated benefits including improved myocardial calcium handling, enhanced cardiac function, reversal of ventricular remodeling, and reduced arrhythmias, laying the foundation for clinical translation [135].

PLN is a protein located on the sarcoplasmic reticulum membrane of cardiomyocytes and serves as a key regulator of cardiac function. It modulates calcium cycling by interacting with the sarcoplasmic reticulum calcium ATPase (SERCA2a), thereby influencing cardiac contractility. Studies reveal that under heart failure conditions, PLN undergoes degradation via Ser^16^ phosphorylation-mediated ubiquitination, with oligoubiquitination at the Lys^3^ site being critical for degradation initiation. The interaction between SERCA2a and PLN inhibits this degradation process. However, during heart failure, SERCA2a expression is transcriptionally downregulated, causing it to lose its inhibitory effect on PLN degradation [136]. These findings suggest that regulating PLN Ser^16^ phosphorylation and Lys^3^ ubiquitination processes, or maintaining SERCA2a expression to enhance its interaction with PLN, may represent potential therapeutic targets for heart failure [137].

Furthermore, Yu et al. [138] observed significantly elevated glycosylphosphatidylinositol-specific phospholipase D1 (GPLD1) expression in cardiac tissues from heart failure patients and mouse models. Myocardially specific knockout of GPLD1 (GPLD1-cKO) exacerbated TAC-induced cardiac dysfunction and myocardial hypertrophy, and fibrosis in patients with heart failure and mouse models. However, an enzyme activity-deficient mutant (GPLD1H133N) failed to exert protective effects, confirming that the cardioprotective function of GPLD1 depends on its intact enzyme activity [138]. This study clarifies the potential of GPLD1 as a novel therapeutic target for HF.

Adenylate cyclase 6 (AC6) is the predominant adenylyl cyclase subtype in mammalian cardiomyocytes, playing a critical role in the pathophysiology of HF by regulating cAMP signaling and calcium homeostasis. Studies reveal that AC6 expression and function are significantly reduced in heart failure, leading to disrupted cAMP signaling and calcium homeostasis. Therefore, AC6 supplementation holds promise as a potential therapeutic strategy [139].

## 7. Summary and Outlook

As the terminal stage of various cardiac diseases, its global prevalence continues to rise with significant demographic disparities. Increasing incidence among both younger and older populations imposes a heavy burden on public health systems. Treatment must account for the acute or chronic nature of onset, the location of pathology, and LVEF, with distinct clinical characteristics and therapeutic strategies for different subtypes. Pathophysiological mechanisms involve multi-system interactions including neurohumoral activation, myocardial remodeling, inflammatory and immune dysregulation, and energy metabolism disorders, offering diverse avenues for therapeutic target development. Current treatment has evolved into personalized precision strategies: HFrEF in chronic heart failure centers on quadruple therapy, while HFmrEF and HFpEF emphasize combination therapy based on SGLT2i. Acute heart failure prioritizes hemodynamic stabilization and early initiation of novel drug therapies, significantly improving patient outcomes. However, challenges remain, including insufficient targeted therapies for HFpEF.

Future research should focus on the following areas: (1) Conducting in-depth investigations into the mechanisms of heterogeneity in HFpEF to advance the development of targeted therapies based on molecular phenotypes; (2) Conducting independent, large-scale RCTs for HFmrEF and the various subtypes of HFpEF to identify optimal treatment pathways; (3) Integrating novel biomarkers (such as circulating microRNAs and metabolomic profiles) with wearable digital health technologies to enable early screening, remote monitoring, and personalized risk prediction for HF; (4) Optimize multidisciplinary collaboration models and design specialized clinical trials for specific populations, such as the elderly and those with multiple comorbidities, to fill gaps in the evidence base. Only through full-chain innovation—spanning from “mechanism elucidation” to “clinical validation” and “comprehensive management”—can the HF care model truly shift from reactive treatment to proactive prevention and comprehensive rehabilitation, thereby reducing the global disease burden.
